# Investigation of nanotexture fabrication by magnetic field assisted ultra-precision diamond cutting

**DOI:** 10.1371/journal.pone.0326046

**Published:** 2025-06-16

**Authors:** Tao He, Muhammad Rehan, Linhe Sun, Jiuxing Tang, Denghui Li, Lulin Chen, Lingling Chen, Suet To, Wai Sze Yip

**Affiliations:** 1 State Key Laboratory of Ultra-precision Machining Technology, Department of Industrial and Systems Engineering, The Hong Kong Polytechnic University, Kowloon, Hong Kong SAR, China; University of Sharjah, UNITED ARAB EMIRATES

## Abstract

Ultra-precision machining (UPM) is crucial for producing parts with functional surfaces featuring nano textures, yet it faces challenges in generating such textures. This paper explores the potential of magnetic field-assisted UPM to overcome these challenges by leveraging magnetophoresis to generate nanotextures and thoroughly investigating the importance of cutting velocity on magnetophoresis in diamond cutting. Experimental results from cutting force, surface profile, surface topography, and atomic force microscopy images demonstrate that magnetic fields enable nanotexture generation on aluminum alloys surfaces in diamond cutting. Also, increasing cutting speed in diamond cutting under a magnetic field enhances magnetophoresis. This study highlights the advantages of integrating magnetophoresis for advanced nanotexture fabrication in UPM and emphasizes strategies for control cutting speed to achieve nanotextures.

## Introduction

Ultra-precision machining (UPM) technology, as one of the promising technologies to achieve high-precision machined surfaces and high-integrity free-form surfaces, can meet the accuracy requirements of various advanced functional structures between micron and nanometer surface roughness [1–3]. This technology is essential to fabricate micro- and nanostructures that exhibit unique surface properties, such as superhydrophobicity for reduced surface wetting [4], adhesion [5], and directionality to control cell migration [6,7]. Generally, the single-point diamond cutting technique in UPM technology offers a high degree of machining freedom, utilizing tool tips with radii ranging from a few millimeters, and is commonly used to machine various nanostructures [8,9]. However, due to the diverse properties of materials, few combine high strength and make challenges for UPM [10]. Materials used in semiconductor and optoelectronic industries, such as silicon, silicon carbide, and tungsten carbide, are characterized by their hardness, brittleness, and low fracture toughness, which can result in premature chipping during the machining of intricate profiles or nanostructures and nanotextures [11–13]. Conversely, high-strength alloys, including titanium and nickel-based superalloys, present a different set of challenges due to adhesive wear mechanisms. The chemical affinity between diamond tools and ferrous materials leads to heat generation and diamond tool degradation through graphitization [14,15].

Multi-layered micro-structures and complex geometries for functional surfaces with stringent accuracy requirements, UPM encounters bottlenecks in terms of process efficiency and machining complexity. The need for precise layer alignment, avoidance of interfacial damage, and maintaining nanoscale tolerances demands advanced process control. Apart from the process control, the fabrication of microstructures with nano-textures or hierarchical nano-microstructures using UPM presents significant challenges. First, material swelling in UPM is an issue because materials can expand during diamond machining at the microscale due to thermal expansion, making nano-scale textures difficult due to deformation. Second, the inherent constraint of edge radius of diamond tools, usually in millimeter scale, presents a significant challenge for nano-feature fabrication. This is because the edge sharpness necessary for precise nano-scale machining is beyond the capability of standard tools with such relatively large radii, thereby limiting the creation of detailed nano-textures effectively. Finally, the minimum cutting depth issue arises, as attempting to cut below the microscale threshold frequently results in ploughing rather than actual material removal, defeating nano-machining [16,17]. These challenges require comprehensive process control and advanced technology to overcome, emphasizing the advancement in UPM for those nano-textures or hierarchical nano-microstructures for functional surfaces.

Researchers have demonstrated that integrating magnetic fields into UPM significantly increases surface integrity and mitigates swelling issues, crucial for the fabrication of nano-textured and microstructures. This innovative approach, as attested by extensive research, delivers enhanced surface quality with a substantial reduction in surface roughness. The stabilizing effect of magnetic field is particularly notable in mitigating chatter and enabling precise control in material removal, thereby increasing capability of fabrication in nano-microstructures or nanotextures by UPM. Studies by Smirnov and Guo et al. [18,19] have revealed that the application of a magnetic field during machining significantly influences the material response, altering its strain energy state, and inducing surface hardening. This phenomenon, coupled with changes in subsurface plastic deformation, contributes to the formation of stable machining regimes, yielding improved surface integrity and the potential for specific functional surface characteristics. Xiao et al. [20] reported a significant improvement in surface finish when magnetic field-assisted UPM was applied to nickel-based superalloys, observing a reduction in surface roughness from 23 nm to 13 nm. This technique not only enhances surface quality but also influences the mechanical properties of the machined surface, as demonstrated by an increase in hardness and ductility. Li et al. [21] further validated the stabilizing effect of magnetic fields on the diamond turning process, observing chatter reduction and a notable decrease in surface roughness by approximately 39%, highlighting the potential for enhanced control and precision. Guo et al. [22] through micro-indentation and ultra-precision micro-cutting experiments, provided evidence of the magnetic field ability to modify the plastic behavior of materials, reducing the stacking effect and expanding the plastic deformation zone. Actually, magnetic field-assisted UPM utilizes the principles of physics and magnetism to revolutionize metal machining, especially enhancing the production of nano-textured and hierarchical nano-microstructures. This method presents multiple benefits: it induces eddy currents in conductive materials, as demonstrated by Yip et al. [23] in single point diamond turning of titanium alloys, utilizing Lorentz forces and eddy current damping effects for vibration reduction, thus enhancing process stability and surface quality. The interaction of the magnetic field with material properties, through the modification of electron-dislocation dynamics, facilitates surface hardening, thereby diminishing friction and tool wear, according to research findings. The formation of magnetic dipoles facilitates heat dissipation, thereby reducing surface swelling and adhesive wear, as demonstrated by Khalil et al. [24] through diminished tool wear and improved cutting quality. Yip et al. [25] highlighted the substantial enhancement in precision, attaining an accuracy exceeding 98%, and the reduction in surface deformation, enabling the controlled formation of micro-nanostructures through magnetophoresis. These collective insights illustrate the magnetic field capacity to surpass conventional UPM constraints, incorporating vibration mitigation, material properties improvement, and thermal dissipation for potentially fabricating complicated nano-structured geometries or nanotextures.

Magnetophoresis emerges as a promising technique within UPM to elevate surface precision and exert control over material removal processes, enabling the fabrication of intricate microstructured metallic surfaces adorned with defined patterns. This phenomenon, based on the directed movement of suspended metallic particles in response to magnetic field gradients, uses magnetic forces and torques to precisely position or transport particles in accordance with magnetostatic principles [26]. Yip et al. [27] demonstrated this by using a magnetic field perpendicular to the cutting path during aluminum alloy machining with a single-point diamond tool, resulting in an ordered array of superhydrophobic microstructures. The advantage of this technique derives from its simplicity, which eliminates the need for complex machining infrastructures and provides a potentially more efficient route to creating hydrophobic metal surfaces than traditional methodologies. Actually, the integration of magnetophoresis within UPM presents a complex interplay between magnetoplastic effects, surface quality enhancement, and precise micro- nanostructure and nanotexture fabrication, necessitating scientific investigation to ensure process control and reliability. The integration of magnetophoresis within UPM introduces a sophisticated interplay involving magnetoplastic phenomena, surface quality improvements, and precise fabrication, necessitating in-depth research for controlled and reliable processes. Guo et al. [28] have highlighted the anisotropic impact of magnetic fields on material behavior, with perpendicular alignment reducing subsurface damage and enhancing crystal structure, yet this effect is dynamically influenced by cutting speed, which mitigates magnetoplasticity and material flow. Insights from Pamme et al. [29] on magnetic particle separation show the significance of field strength and flow dynamics, paralleling the importance of magnetic field strength and cutting speed in dictating particle movement.

Prior studies have not sufficiently addressed the specific influences of these variables on magnetophoresis, despite their essential roles in achieving the intricate nano-textures and hierarchical nano-microstructures demanded by advanced functional surfaces. This study fills a critical gap in literature by undertaking a thorough examination of UPM parameters, particularly cutting speed and magnetic field intensity, impact on magnetophoresis, a phenomenon crucial for enhancing the precision and efficiency of nanotexture fabrication in UPM. By investigating the interplay between magnetic field intensity, cutting parameters, and the surface quality, a deeper understanding and optimized application of UPM techniques can contribute to nanotextured surfaces.

## Theory

### Plastic side flow

**Fig 1:**
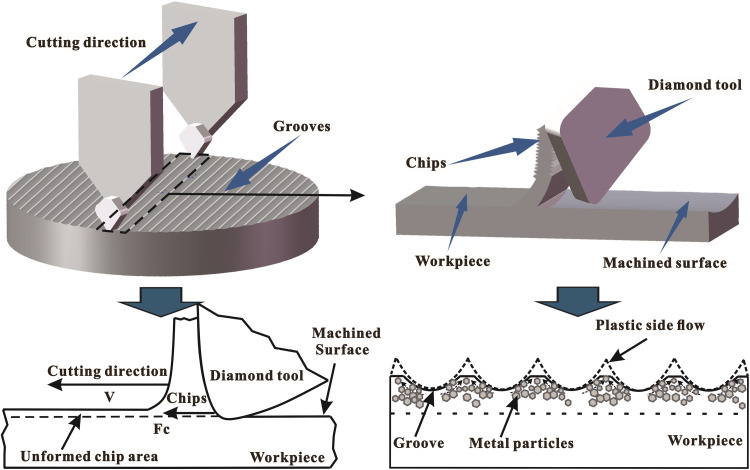
The plastic side flow phenomenon and its effect on the surface topology of machined surface.

In UPM, grooving with a single-point diamond tool often results in plastic side flow on both sides of the groove. This phenomenon occurs when material near the cutter head is displaced to the sides and flows to the free surface due to thrust forces. As a result, the grooves or ridges formed by plastic side flow increase the measured surface roughness. Extensive research has been conducted on plastic side flow in ultra-precision machining [30]. Show et al. [31] highlighted that this phenomenon is more pronounced at smaller depths of cut and higher cutting speeds. They established a relationship between plastic side flow material buildup and average cutting energy, noting that higher average cutting energy leads to more pronounced plastic flow at the cutting edge. Generally, material buildup and flow due to plastic side flow depends on the material’s strength and ductility. Wang et al. [32] observed material surface buildup due to plastic flow when micromachining 6061-aluminum alloy with diamond tools, indicating that the alloy’s strength and ductility contribute to the occurrence of plastic side flow phenomena. To evaluate the intensity of plastic side flow, we calculated the average cutting energy during grooving using the following formula [33]:


$$W=F_{c}v_{c}t=F_{c}s$$ (1)

where W is the average cutting energy, $F_{c}$ is the cutting force, $v_{c}$ is the cutting speed, t represents the average machining time per unit length of cutting-edge during machining process, and s represents unit length of cutting, as shown in Fig 1.

In magnetic field-assisted UPM, the dynamics of plastic side flow are modified by introducing an external magnetic field, utilizing the concept of magneto-plasticity, where the material’s plastic deformation behavior is influenced by magnetic forces. The magnetic field induces the movement of metal particles within the material, aligning them in a controlled manner. This interaction creates a coupling effect, where magnetic forces guide the flow of material during machining, offering several advantages, especially enhanced surface finish through predictable material displacement and improved dimensional accuracy for the precise machining of nanostructures.

### Magnetophoresis in diamond cutting

Magnetophoresis, a crucial phenomenon for explanation in this research, entails the directed migration of magnetically responsive particles within metallic matrices towards regions with magnetic field strength gradient [26]. This process is underpinned by the synergistic application of Stokes’ Law and the Lorentz force law. Stokes’ Law, a theory of fluid mechanics, provides the velocity dynamics of particles suspended in a viscous environment, pertinent to the machining fluids employed. Conversely, the Lorentz force law, detailing the force experienced by charged particles traversing a magnetic field, is crucial in magnetophoresis, particularly for micro- and nano-debris generated during diamond cutting processes. These charged particles, suspended in the cutting medium, experience a magnetophoretic force, mathematically represented as a function of particle properties, magnetic field intensity, and its spatial gradient, as per equations derived in seminal works [34,35]. This force, intricately balanced with the viscous resistance described by Stokes, governs the unique particle movement, thereby influencing the microenvironment and the quality of the machined surface at the micro- and nano-scale, highlighting the scientific depth and practical implications of this study. In magnetophoresis, charged particles (micro/nano debris within the viscous medium from diamond cutting) in an applied magnetic field are subjected to a force-the magnetophoretic force, as expressed in the formula,


$$F_{m}=V_{p}\mu_{0}\chi H\nabla H$$ (2)

where $V_{p}$ is the volume of the particle, χ is its magnetic susceptibility, $\mu_{0}$ is the vacuum permeability, H is the intensity of the magnetic field, and ∇H is the gradient of the magnetic field. Simultaneously, in UPM of aluminum alloy specimens, the material is extruded and deformed due to cutting forces and heat. This machining area is subjected to magnetophoresis under the influence of a magnetic field, which enhances the material’s plasticity and affects the magnetophoresis phenomenon. During the UPM process, micro and nano metal particles are generated and move within this medium. Consequently, the material itself behaves like a viscous medium, and its viscosity influences the velocity of the particles. This velocity can be controlled using Stokes’ Law, expressed as:


$$F_{d}=6\pi \eta vR$$ (3)

$$v=\left(\frac{1}{18}\epsilon \mathrm{\backslash rightleft}(p_{p}-p_{f}\right)g^{2}$$ (4)

where R is the radius of the sphere, η is the viscosity coefficient of the liquid, v is the moving particle speed, ϵ is the dynamic viscosity of medium, $p_{p}$ and $p_{f}$ are the densities of particles and fluid respectively, g is the gravitational constant. Stokes’ Law supports control of metal path movements and the corresponding magnetic forces on the machined surface due to viscous resistance. The synthesis of these theories will explain the movement of magnetic particles and the nanoscale footprint left due to the magnetophoresis effect in diamond cutting.

**Fig 2:**
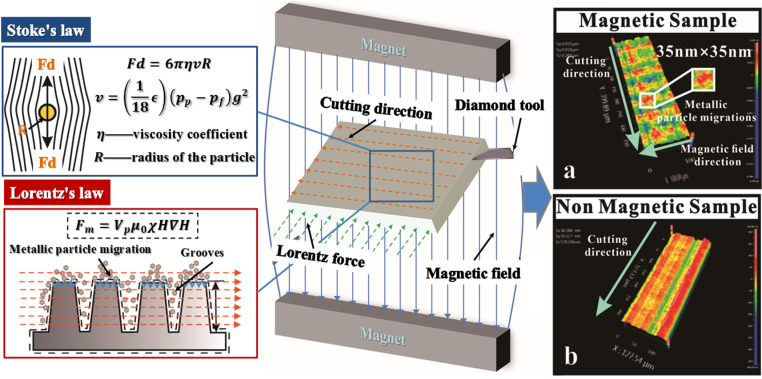
The magnetophoretic phenomenon and the surface topology of titanium alloy surface generated in diamond cutting (a) in a presence of magnetic field and (b) in an absence of magnetic field *The images quoted above are similar but not identical to the original images and have been rearranged. The images are from [25,27].

Extensive research [36,37] shows the significant role of magnetic fields in UPM, revealing dual mechanisms that enhance machining efficiency and precision. Firstly, by optimizing heat distribution and mitigating thermal gradients within the cutting zone, magnetic fields resolve challenges encountered when machining hard-to-machine materials, thereby improving overall machining performance. Secondly, the magnetic field ability to induce eddy current damping effect and convert it into an equivalent damping force via the Lorentz force effectively reduces system vibrations, leading to heightened machining accuracy and efficiency. In magnetophoresis-assisted diamond cutting, a specialized magnetic fixture integrated into UPM creates a controlled magnetic field in the machining area. As the process progresses, a metallic fluid mixed with particles appears. The migration of these particles, directed by the magnetophoretic force, follows Stokes’ Law, with their trajectory and velocity determined by the dynamic viscosity of the fluid, which is influenced by the interaction of diamond cutting speed and applied cutting forces. This intricate interplay not only reduces heat and vibration but also contributes to good surface finish and the controlled creation of micro- and nanotextures, as shown in Fig 2.

### Experimental setup

**Fig 3:**
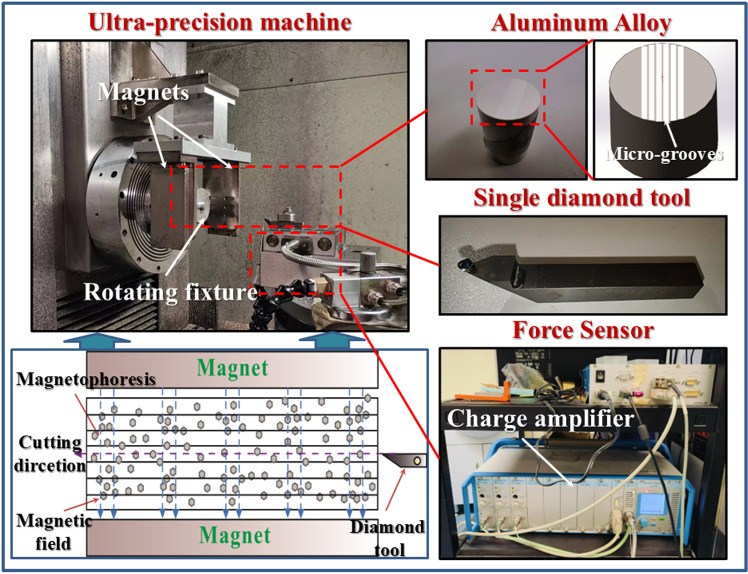
The experimental setup. *The images quoted above are similar but not identical to the original images and have been rearranged. The images are from [27].

This experimental investigation focuses on demonstrating generation of nanotextures on a metal surface by diamond cutting using magnetophoresis through the application of a magnetic field. The study explores the interplay between cutting velocity and magnetic field intensity on the generation of nano-textures. The experimental design includes two sets: varying cutting velocity at a constant magnetic field and vice versa, to confirm and better understand their effects on groove formation and surface nano-texturing. Two sets of experiments were conducted, wherein the aluminum workpiece was uniformly machined with an array of 500 grooves covering the surface. The 6061-aluminum alloy specimen used for the experiment was a cylinder with a diameter of 11.75 mm and a length of 21 mm. A single point diamond tool with a tool radius of 1.018 mm was used for surface flat cutting and then a single point diamond tool with a tool radius of 0.909 mm was used for cutting groves. One set of experiments includes varying cutting velocity (100–250 mm/min) at a fixed magnetic field intensity of 0.02 T, while another maintains a constant velocity of 100 mm/min, altering the magnetic field intensity from 0 to 0.03 T, with a constant depth of cut and groove width. The cutting fluid is Isomeric alkane fluids (Isopar H). The investigation employs an Atomic Force Microscopy (AFM), Scanning Electron Microscopy (SEM), and a high-precision optical profiler (Zygo) to scrutinize the nano-textured surfaces, confirming the magnetically induced surface features. Cutting force measurements by a Kistler dynamometer (type: 9256C2) with a sampling frequency of 1 kHz, captured in real-time, further explores the mechanical dynamics during the induction of surface-texturing. These experiments comprehensively illustrate and confirm the nano-texturing potential and quality enhancements achievable through controlled magnetic field effects in the diamond cutting process, providing insights into the magnetophoresis assisted surface texturing process in UPM. The experimental setup is shown in Fig 3, with the detail shown in Table 1.

**Table 1:** The machining parameters in experiments.

Sample	Depth of Cut (μm)	Distance between each groove (mm)	Cutting velocity (mm/min)	Magnetic field intensity (T)	Number of grooves
**1**	5	0.01	100	0.02	500
**2**	5	0.01	150	0.02	500
**3**	5	0.01	200	0.02	500
**4**	5	0.01	250	0.02	500
**5**	5	0.01	100	0	500
**6**	5	0.01	150	0	500
**7**	5	0.01	200	0	500
**8**	5	0.01	250	0	500
**9**	5	0.01	100	0.01	500
**10**	5	0.01	100	0.02	500
**11**	5	0.01	100	0.03	500

## Results and discussion

### Cutting force

**Fig 4:**
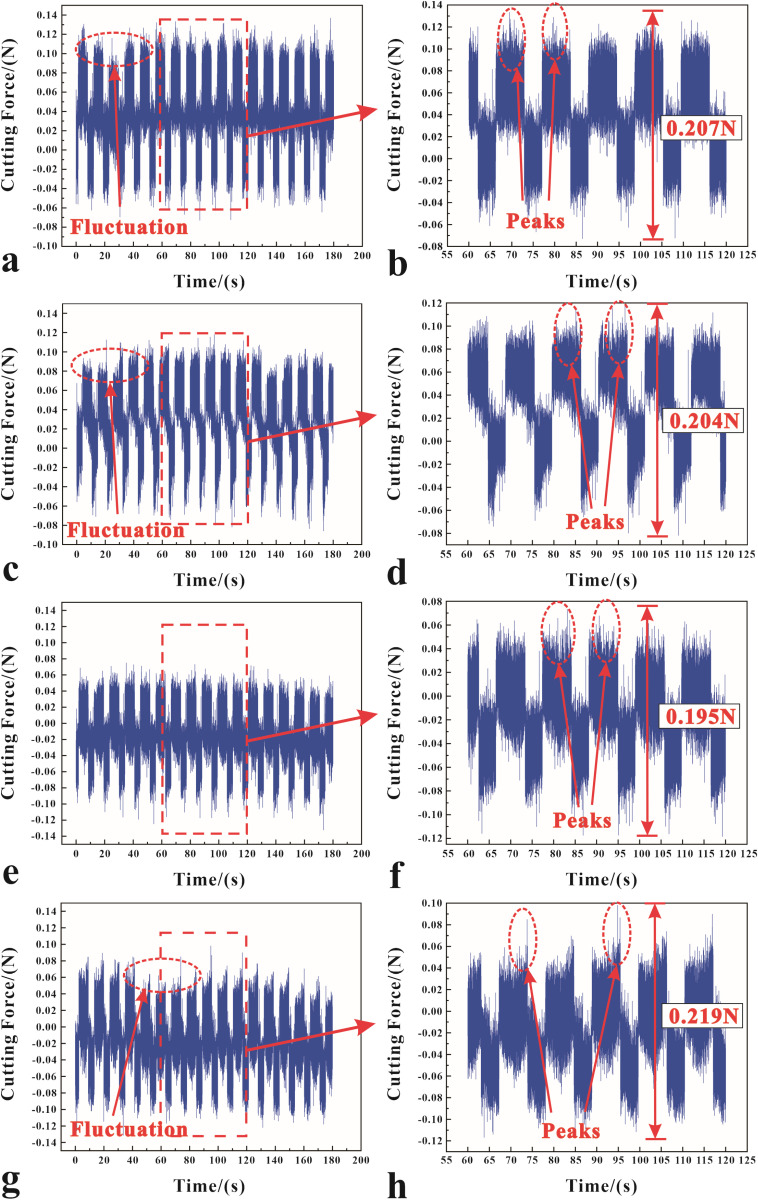
The cutting forces at different magnetic field intensities: (a-b) 0 T, (c-d) 0.01 T, (e-f) 0.02 T, and (g-h) 0.03 T.

Magnetophoresis significantly influences the dynamics of diamond cutting processes by altering the distribution and movement of metal fluid at the cutting interface. Upon application of a magnetic field, suspended magnetic particles within the fluid align and migrate along field lines, enhancing fluid flow dynamics around the cutting tool. This improvement in lubrication, as depicted in Fig 4, is particularly evident at a critical magnetic field strength of 0.02 T. At this threshold, the magnetic force optimizes fluid dynamics, creating a more effective lubricating barrier that reduces direct tool-workpiece contact and thereby cutting forces, which are observed to decrease to approximately 0.195 N, indicating of reduced friction and controlled material displacement. The fluid enhanced behavior ensures a consistent and stable cutting process, critical for UPM to achieve uniform surface finishes. However, beyond 0.02 T, the benefits of magnetophoresis can diminish, potentially disrupting fluid dynamics due to altered viscosity or magnetic field-induced flow disturbances, impacting heat dissipation and surface finish negatively. As the magnetic field increases, it can alter fluid behavior, potentially causing instability in the magnetophoresis effect. At 0.03 T as shown in Figs 4g-h, it indicates increased force fluctuations, signaling reduced machining quality due to instability.

The fluctuation of the cutting force curve in Fig 4 further validates the above analysis and conjecture. When the magnetic field strength is 0 T and 0.01 T, the diamond tool and the cutting material need some time to adapt to reach a stable cutting process, which is shown in the image as the initial curve fluctuation transition to a relatively smooth cutting state, and in the subsequent processing will also be accompanied by a slight fluctuation of the cutting force. For the cutting force curve with a magnetic field strength of 0.03 T, the fluctuation pattern of the cutting force is even more unstable. From Figs 4G-H, the pattern of peak fluctuation of the cutting force is the largest.

**Fig 5:**
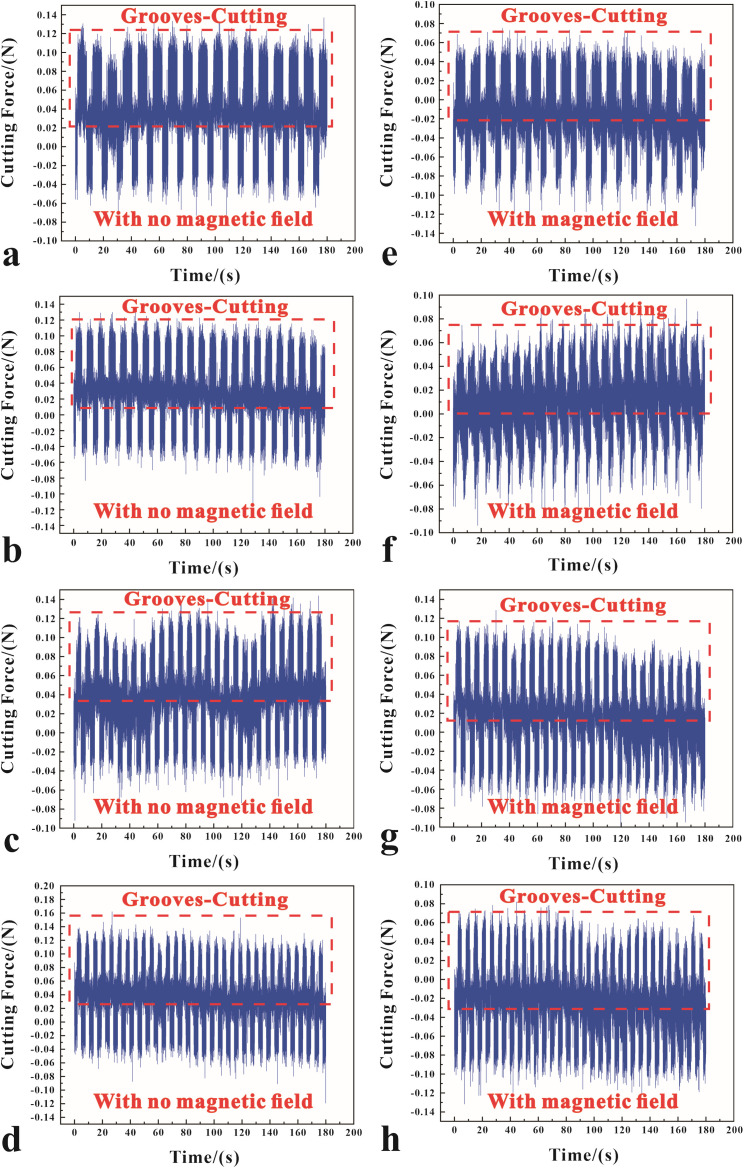
Cutting forces at different cutting velocities with no magnetic field: (a) 100 mm/min, (b) 150 mm/min, (c) 200 mm/min, (d) 250 mm/min, and with a magnetic field strength of 0.2T: (e) 100 mm/min, (f) 150 mm/min, (g) 200 mm/min, (h) 250 mm/min.

As illustrated in Fig 5, a significant relationship is found between cutting speeds under a magnetic field and a gradual increase in cutting force. This rise in cutting force causes a noticeable rise in force variability, indicating a more dynamically challenging machining environment. However, the strategic integration of magnetic fields into this process creates a new dynamic that draws on the fundamental principles of the Lorentz force and magnetophoresis. This strategic application not only mitigates the negative effects of increased cutting forces, but it also uses these physical phenomena to exert a stabilizing influence, potentially guiding particle movement and force distribution in a way that improves the precision and control of the machining process. Our findings highlight a new paradigm in ultra-precision machining, in which magnetic fields act as a regulatory force, optimizing machining parameters and allowing the creation of nanotextures in diamond cutting with unprecedented accuracy.

Comparing the data curves in Fig 5, the magnetic field is significant for the reduction of cutting forces. At high cutting speeds, such as 250 mm/min, the reduction in cutting force by approximately 0.7 N demonstrates the controlled particle movement, which reduces the disruptive forces that typically degrade surface finish. The combined effects appear when the cutting velocity is optimized to 100 mm/min and the magnetic field strength is 0.2 T. The magnetically influenced, more ordered particle flow reduces cutting forces and significantly reduces vibrations, allowing for grooves with an exceptional surface finish.

**Fig 6:**
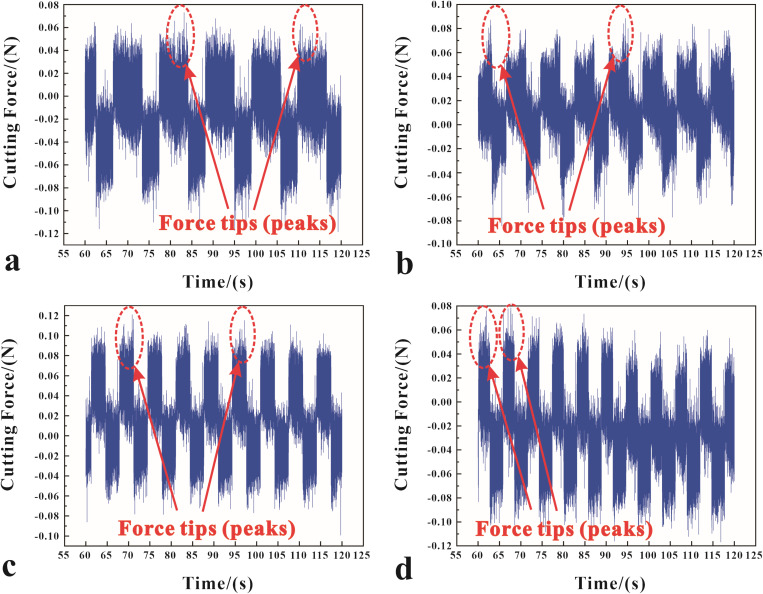
The cutting forces at different cutting velocities under a magnetic field strength of 0.02 T: (a) 100 mm/min, (b) 150 mm/min, (c) 200 mm/min, (d) 250 mm/min.

To better understand the role of magnetophoresis in diamond cutting at various speeds, an in-depth analysis of cutting forces at different speeds under a magnetic field intensity of 0.02 T was conducted, with the results illustrated in Fig 6. The data graph shows that the peak cutting force’s tip amplitude increases with an increasing speed up to 200 mm/min, then drops again at 250 mm/min. The increase from 100 mm/min to 200 mm/min suggests more dynamic movement of metallic particles and the formation of nanotextures, highlighting the interaction between particle dynamics and the cutting process within this speed range. The forces at work, such as the Lorentz force and material adhesion, are seen to have an intricate influence on cutting force dynamics. Mansori et al. [38] concluded that the magnetic effect enhances the ductility of the material, i.e., the mobility of the internal material particles, during higher cutting speeds. This phenomenon exhibited lower subsurface shear flow during grooving and the effect was maximized at high cutting speeds and shallow feeds. Stokes’ Law, which relates particle resistance to fluid viscosity, is important here.

### Surface topology

**Fig 7:**
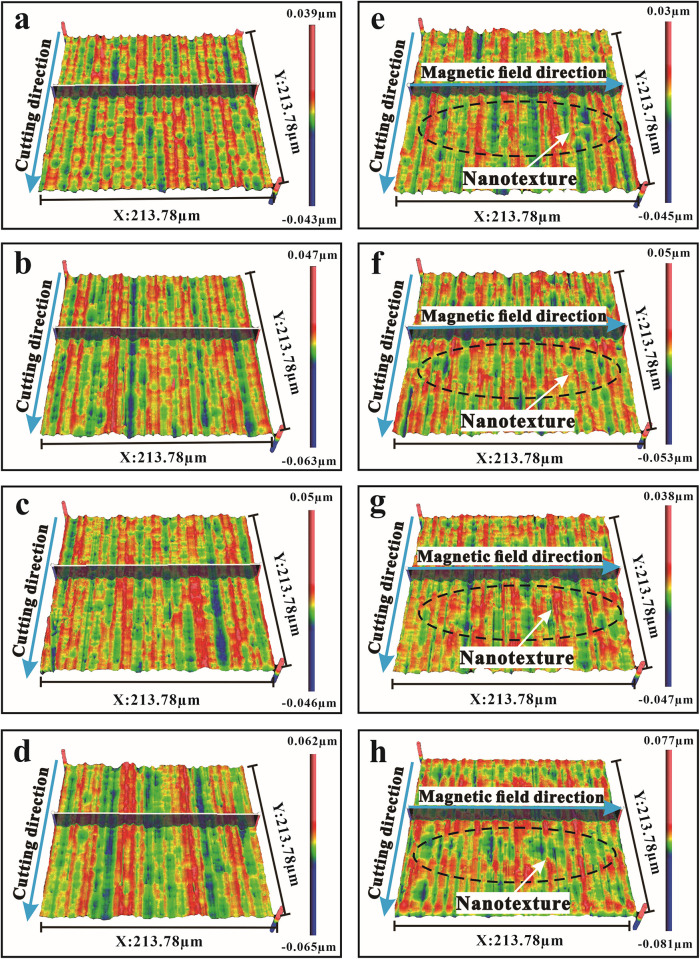
3D Surface topographies at different cutting velocities with no magnetic field: (a) 100 mm/min, (b) 150 mm/min, (c) 200 mm/min, (d) 250 mm/min, and with a magnetic field strength of 0.2T: (e) 100 mm/min, (f) 150 mm/min, (g) 200 mm/min, (h) 250 mm/min.

Fig 7 demonstrates the capability to create nanotextured surfaces through magnetic field-assisted diamond cutting employing magnetospheric. The machined surface without a magnetic field exhibits a surface profile with longitudinal ridges and valleys caused by plastic flow perpendicular to the cutting direction. In contrast, the surface machine with magnetic field assistance shows a different pattern. The magnetic particles migrate in accordance with the magnetic field, covering the bar-like features with nanotextures. This combination of forces and material flow produces a more complex and potentially beneficial surface texture, demonstrating the capability of magnetic field-assisted machining to control and engineer surface features at the nano scale, even if it appears to compromise traditional surface quality measures at high cutting speeds.

Figs 7A-D illustrates the surface profile of aluminum alloy machined at varying cutting speeds without the influence of a magnetic field, revealing a distinct pattern change. At a cutting speed of 100 mm/min, the surface remains relatively flat despite the presence of surface irregularities or peaks, suggesting a balance between material removal and surface deformation at this speed. However, as the cutting speed increases, the surface profile changes significantly, with peaks becoming more pronounced and surface flatness varying considerably. According to Eq. 1, the average cutting energy is directly related to the cutting force, which increases with cutting speed. As the cutting speed rises, the average cutting energy also increases, enhancing the plastic lateral flow effect. This causes more metal particles to move under the cutting force. On the other hand, without a magnetic field, the magnetophoretic effect is absent, meaning that magnetophoresis does not contribute to the formation of nanotextures in diamond cutting. The observed changes in surface nanotextures at higher cutting speeds, as shown in Fig 7, are therefore solely due to mechanical effects rather than any magnetophoretic influence. This highlights the critical role of a magnetic field in enabling magnetophoresis, which is absent in this scenario. This observation is consistent with the findings of Wang et al. [30] in their study on ultra-precision grooving of 6061 aluminum alloy. They discovered that increasing the cutting speed which is indirectly related to cutting speed in terms of material removal intensity, causes an increase in cutting forces. In the case of machining 6061 aluminum alloy, which is known for its ductility and moderate hardness, increased cutting forces intensify the material plastic deformation. Due to the high forces used during the cutting process, the material tends to flow plastically, particularly along the groove sides. This plastic side flow effect becomes more pronounced at higher speeds, as the energy imparted to the material increases, causing it to deform more easily. As a result, material accumulates along the groove edges, creating more prominent peaks and valleys on the surface. This effect is especially noticeable when the cutting speed reaches 250 mm/min, demonstrating the machining process sensitivity to speed in terms of achieving a smooth surface finish for this specific alloy.

Investigating the effect of cutting speed with a constant magnetic field intensity, for 0.02 T, reveals remarkable dynamics, as shown in Figs 7E-F. The magnetic field effect on metallic particle migration may be less significant at slower cutting speeds due to lower cutting forces and potentially less intense plastic deformation. These nano-structures become more visible as the cutting speed increases. This unexpected result suggests that the increased cutting speed encountered in a magnetic field environment improve the plastic deformation process, promoting the formation of nanotextures on the surface of the 6061aluminum alloy. For the surface topology at a cutting speed of 250 mm/min under the magnetic field, it does exhibit obvious distinct nanotextures. The principles of Stokes’ Law and Lorentz’s Force Law can help to explain this unique effect. As the diamond tool operates under increased pressure and load in a magnetic field, metal particles move across the cutting plane. This results in plastic side flow, a phenomenon in which a combination of forces, including the magnetic field force, the electric field force caused by the movement of charged particles, and the Lorentz force, act as a driving force. These forces control the resistance felt by the particles, guiding them along the magnetic field lines. As a result, the particles form nanopatterns on the surface.

### Surface profile

**Fig 8:**
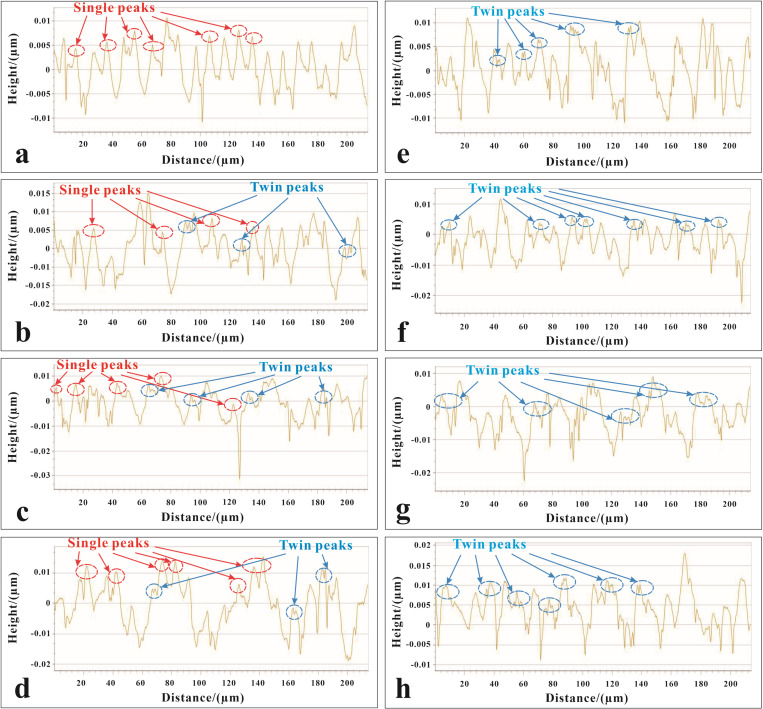
Surface profiles at different cutting velocities without a magnetic field: (a) 100 mm/min, (b) 150 mm/min, (c) 200 mm/min, (d) 250 mm/min, and with a magnetic field strength of 0.02 T: (e) 100 mm/min, (f) 150 mm/min, (g) 200 mm/min, (h) 250 mm/min.

To establish the effect of magnetophoresis in diamond cutting with a magnetic field at 0.02 T, a comparison is made against conditions without a magnetic field. In the absence of a magnetic field, as seen in Figs 8A-D, the aluminum alloy surface is shaped by deformation patterns, showing bar-like structures that caused plastic deformation. The surface has a series of irregular, single peaks with microscale dimensions, which become more unstable with higher cutting speeds. This demonstrates that surface morphology is governed by mechanical forces, with minimal particle guidance. However, Figs 8E-H illustrates a shift when a 0.02 T magnetic field is applied. As the cutting speed increases, the previously singular peaks on the surface transform into well-defined dual peaks. This transformation, which is accompanied by a significant increase in surface pattern regularity and stability, directly links magnetophoresis to particle motion. The presence of a magnetic field allows for more precise groove formation, which reduces surface stress and promotes the formation of consistently structured nanotextures. The findings highlight the critical role of magnetophoresis in refining the diamond cutting process, resulting in improved control and uniformity in the machined surfaces.

### Surface morphology

**Fig 9:**
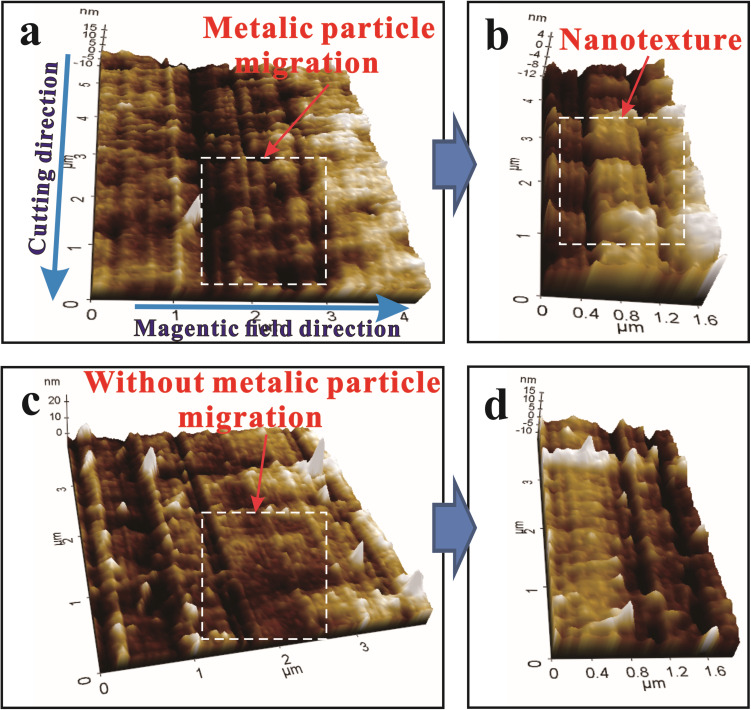
AFM images of machined surface at a cutting velocity of 200 mm/min: (a-b) with a magnetic field strength of 0.02 T, (c-d) without magnetic field.

Fig 9 shows a detailed analysis using atomic force microscopy (AFM) that provides proof for the presence and impact of magnetophoresis in nano surface-texturing during machining of 6061 aluminum alloy. At a cutting speed of 200 mm/min, surfaces machined with and without a magnetic field show distinct differences, emphasizing the role of magnetophoresis in diamond cutting. In Fig 9A, the machined surface processed under a magnetic field shows a clear particle migration phenomenon, with the migration direction aligned horizontally with the applied magnetic field direction. This observation is the result of a combination of forces: the cutting force, which causes deformation, and the magnetic and Lorentz forces, which guide metallic particle movement. The interaction of these forces causes the formation of nanoscale surface features that appear periodically along the grooves. These nanostructures are not random but align with the magnetic field direction; they have a periodicity that suggests a controlled process, which supports the concept of magnetic field assisted surface texturing.

Fig 9B provides a more detailed confirmation, with wave and curl patterns visible on the surface at a nano scale. These patterns show the complex interplay of material deformation and magnetic field-induced particle movement, demonstrating nano-scale texturing capability by magnetophoresis. This is consistent with the findings of Dehghani and Molotskii et al. [39,40], who discovered that magnetic fields intensify dislocation motion in the material, promoting material flow and plastic deformation. This not only improves machining efficiency, but also helps to create unique surface textures, reducing tool wear due to more controlled material displacement. In contrast, Figs 9C-D shows machined surfaces without a magnetic field, revealing a different scenario. The surface features are dominated by plastic flow side extrusion and machining scratches, with no nanostructures indicating magnetophoresis. The absence of these nanostructures confirms that the migration of metal particles, and thus the magnetophoresis effect, is due to the presence of a magnetic field during diamond cutting.

### Chip formation

**Fig 10:**
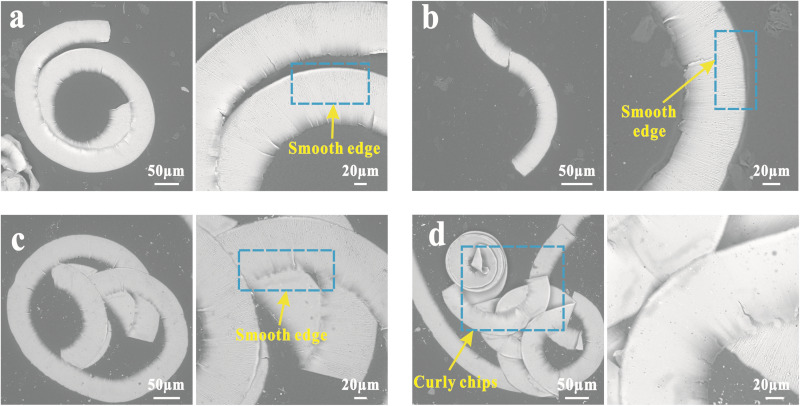
Chip formations at different cutting velocities with a magnetic field strength of 0.2T: (a) 100 mm/min, (b) 150 mm/min, (c) 200 mm/min, (d) 250 mm/min.

Relevant studies [41,42] have proved that chips are produced by complex plowing motions. Material removal occurs due to compressive stress and cutting forces exerted by the tool. The removed chips exhibit different morphological structures due to the distribution of compressive stresses, plastic deformation of the material, and the original properties of the metal, reflecting the overall quality and machining effect of the UPM. Therefore, based on the above analysis of cutting force variation, we collected chips under different cutting parameters, as shown in Fig 10. Under the influence of the magnetic field, the shape of chips is smooth without obvious sawtooth. With the gradual increase in cutting speed, the chips become curlier and more continuous, indicating improved machinability of the material. This observation is consistent with the extensive research conducted by Mansori and Xing et al. [43–45], who investigated the intricate mechanisms involved in magnetic field assisted machining, they investigated the mechanisms involved in magnetic field-assisted machining. Initially, the magnetic field promotes plastic deformation and dislocation movement within the material. This enhancement improves the material ductility and fluidity, lowering the shear strain encountered during cutting. Then, at smaller depth of cut, the increase in cutting speed will lead to an increase in magnetoplasticity, further improving the machinability of the material. This would explain the phenomenon of magnetophoresis to promote the generation of nantextures while ensuring cutting stability and high surface quality.

Magnetic field-assisted ultra-precision diamond cutting is an innovative combination, which can greatly improve the machining accuracy and quality of metal samples, in addition, for the application of such an innovative method can also be extended to the manufacturing field, many researchers have begun to apply the magnetic field-assisted ultra-precision machining method for processing metal samples prepared by 3D printing [46–49]. While using magnetic field-assisted ultra-precision machining, many researchers have also considered other energy fields for ultra-precision machining, such as ultrasonic fields and laser fields [50,51].

## Conclusion

In this study, we demonstrated the significant role of magnetophoresis in fabricating nanotextures during ultra-precision diamond cutting. We also explored how cutting velocity influences the effects of magnetophoresis. By incorporating magnetophoresis, we successfully created nanotextures on material surfaces. Our analysis compares diamond cutting with and without a magnetic field, with key findings including:

1. In diamond cutting without a magnetic field, increased cutting speed leads to higher cutting forces, potentially affecting surface quality. With magnetic field assistance, the cutting forces interact with the magnetic field, inducing magnetophoresis. This effect, combined with plastic side flow lowering, facilitates the formation of nanotextures by directing plastic deformation into controlled patterns, confirming the active role of magnetophoresis in shaping surface features.
2. As cutting speed increases under magnetic field assisted diamond cutting, the interaction between the magnetic field and cutting dynamics intensifies. When cutting speed increases, more single peaks on the grooved surface transform into double peaks due to the migration of metal particles perpendicular to the cutting direction, driven by magnetophoresis.
3. Surface morphology analyses reveal distinct wave and curl patterns, illustrating the role of magnetophoresis. These nano scale patterns indicate how the magnetic field directs material particle flow during cutting.

## Supporting information

S1 File Supporting Data. (RAR)
